# Supplementary material for: Satb1 integrates DNA binding site geometry and torsional stress to differentially target nucleosome-dense regions
Source: Nat Commun. 2019 Jul 19;10:3221. doi: 10.1038/s41467-019-11118-8 (PMC6642133; doi:10.1038/s41467-019-11118-8)
Supplement: Supplementary file 2 — Description of Additional Supplementary Files [file 41467_2019_11118_MOESM2_ESM.pdf]

Description of Additional Supplementary Files

File name: Supplementary Code 1

Description: Custom codes used for a subset of genomics data analysis and Spatiotemporal FRAP data analysis. For details please refer to the "Raw data and codes roadmap" document included with Supplementary Code 1

File name: Supplementary Movie 1

Description: 20 Hz HiLo-TIRF movie and corresponding single molecule trajectories of Satb1 FL

File name: Supplementary Movie 2

Description: 20 Hz HiLo-TIRF movie and corresponding single molecule trajectories of ΔHD

File name: Supplementary Movie 3

Description: 20 Hz HiLo-TIRF movie and corresponding single molecule trajectories of N-C1

File name: Supplementary Movie 4

Description: 20 Hz HiLo-TIRF movie and corresponding single molecule trajectories of N
