# Supplementary material for: Satb1 integrates DNA binding site geometry and torsional stress to differentially target nucleosome-dense regions
Source: Nat Commun. 2019 Jul 19;10:3221. doi: 10.1038/s41467-019-11118-8 (PMC6642133; doi:10.1038/s41467-019-11118-8)
Supplement: Supplementary file 3 — Supplementary Information [file 41467_2019_11118_MOESM3_ESM.pdf]

Satb1 integrates DNA binding site geometry and torsional stress to differentially target nucleosome-dense regions

Ghosh et al.

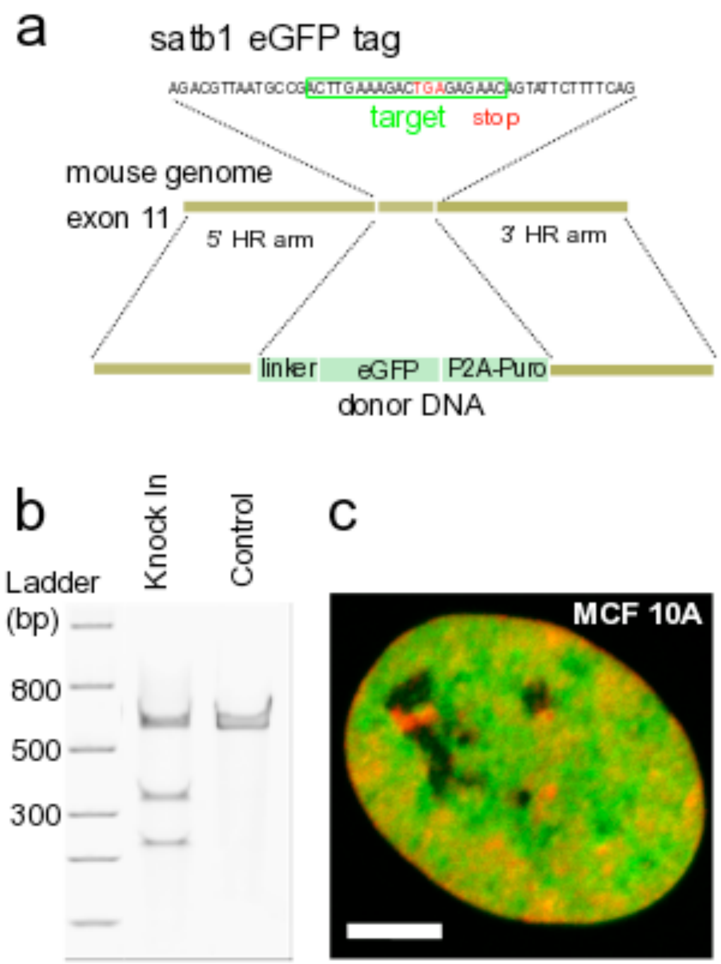

Supplementary Figure 1. a. Schematic representation of CRISPR/Cas9 based eGFP knockin of native Satb1. b. Surveyor assay for verifying double strand breaks generated by sgRNA for C terminal Satb1 eGFP tagging. c. MCF-10A cell stably expressing Satb1 eGFP stained with SIR-DNA stain. Scale bar is 5 μm.

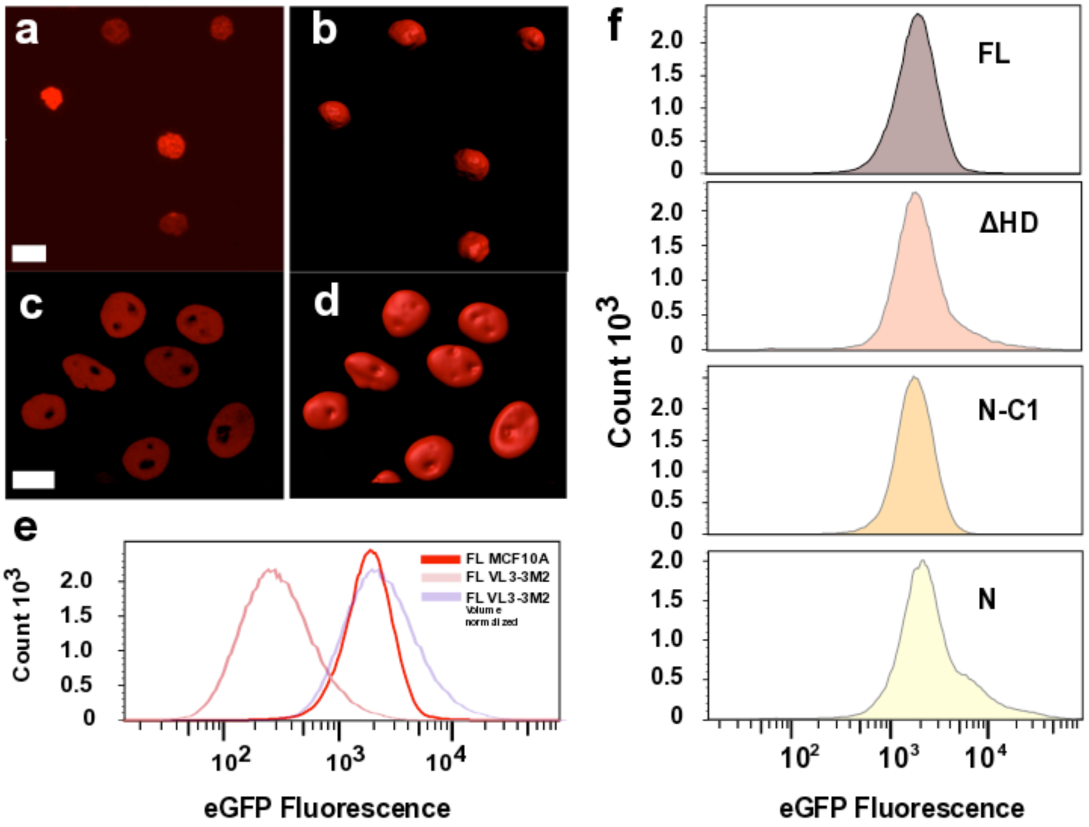

Supplementary Figure 2. Expression levels of Satb1 constructs in stable cell lines.

a, b. Confocal image of Sir-DNA stained VL3-3M2 nuclei (a) and their corresponding 3D volume reconstructions (b). VL3-3M2 has an average nuclear volume of 147± 42. Scale bar is 10μm (n=60).

c, d. Confocal image of Sir-DNA stained MCF10A nuclei (a) and their corresponding 3D volume reconstructions (b). MCF10A has an average nuclear volume of 1131 ±341 μm^3. Scale bar is 20μm (n=50).

e. Flow cytometric analysis of FL Satb1 expression in VL3-3M2 and MCF10A stable cell lines. When normalized for nuclear volume VL3-3M2 and MCF10A cell lines show very similar expression.

f. Representative flow cytometric fluorescence intensity histograms of expression of different Satb1 constructs in corresponding MCF10A cell lines 2 days' post-sorting for an identical fluorescence gate.

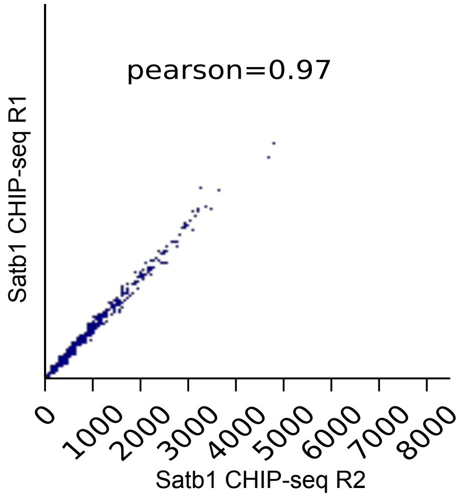

Supplementary Figure 3. Pearson correlation of reads from two independent FL Satb1 ChIP-seq experiments.

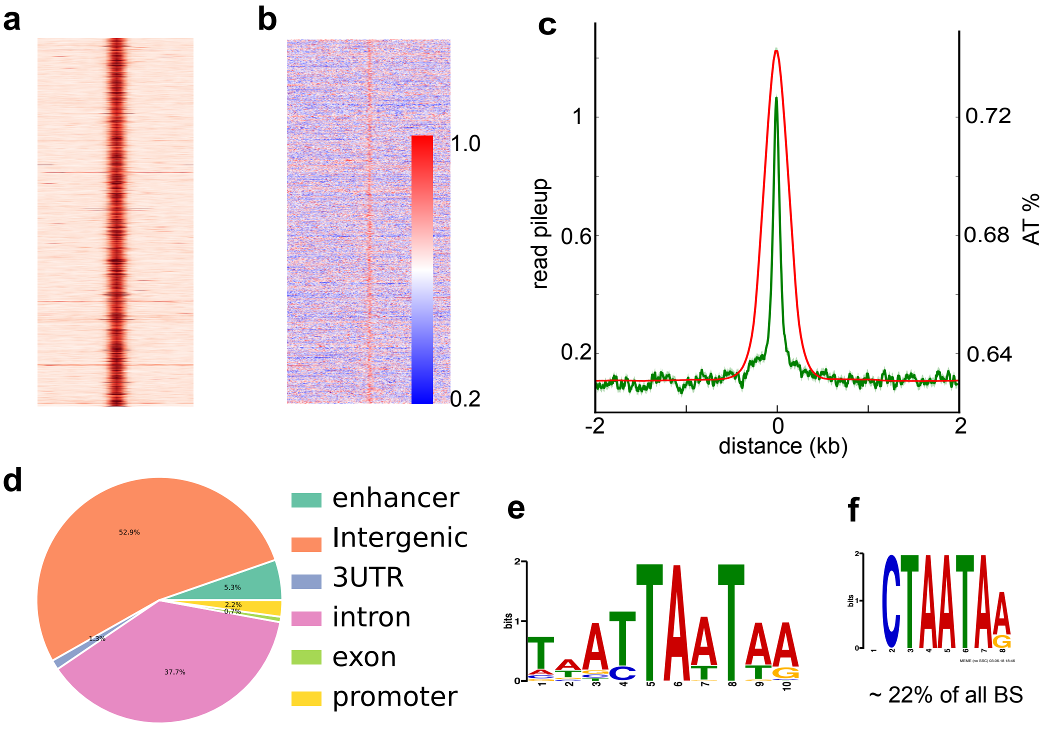

Supplementary Figure 4. ChIP-seq of Satb1 in VL3-3M2 cells. a. Heatmap of ChIP seq signals (read pileups) along a 4kb window centered on binding peaks. b. Heatmap of A/T percentage along a 4kb window around all genomic binding sites of Satb1 c. Overlay of average ChIP-seq signals (green) and A/T percentage along a 4kb window centered on the Satb1 binding sites (red). d. Relative abundance of Satb1 binding sites in different genomic categories. e, f. Two consensus motifs that satisfy 100% (e) and 22% (f) of all binding sites in VL3-3M2.

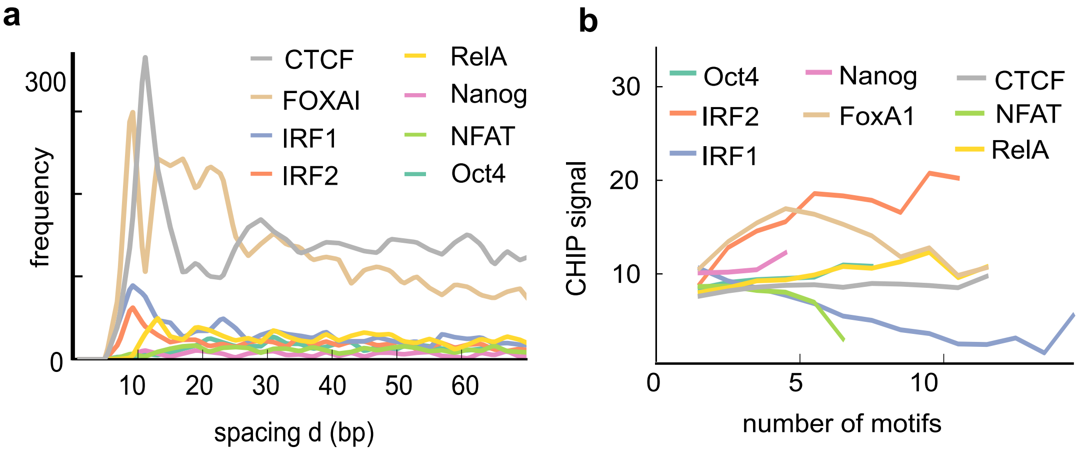

Supplementary Figure 5. Motif spacing and density vs. binding strength in transcription factors. a. Frequency distribution of the distance between motif pairs in a 400bp window, shows no periodic spacing pattern for eight common transcription factors (see methods for data source) with a wide range of genome coverage. b. Mean ChIP-seq signal versus motif density plot shows no signs of cooperativity for the same eight transcription factors. Seven of these transcription factors also prefer to bind to A/T rich DNA (the one exception is CTCF).

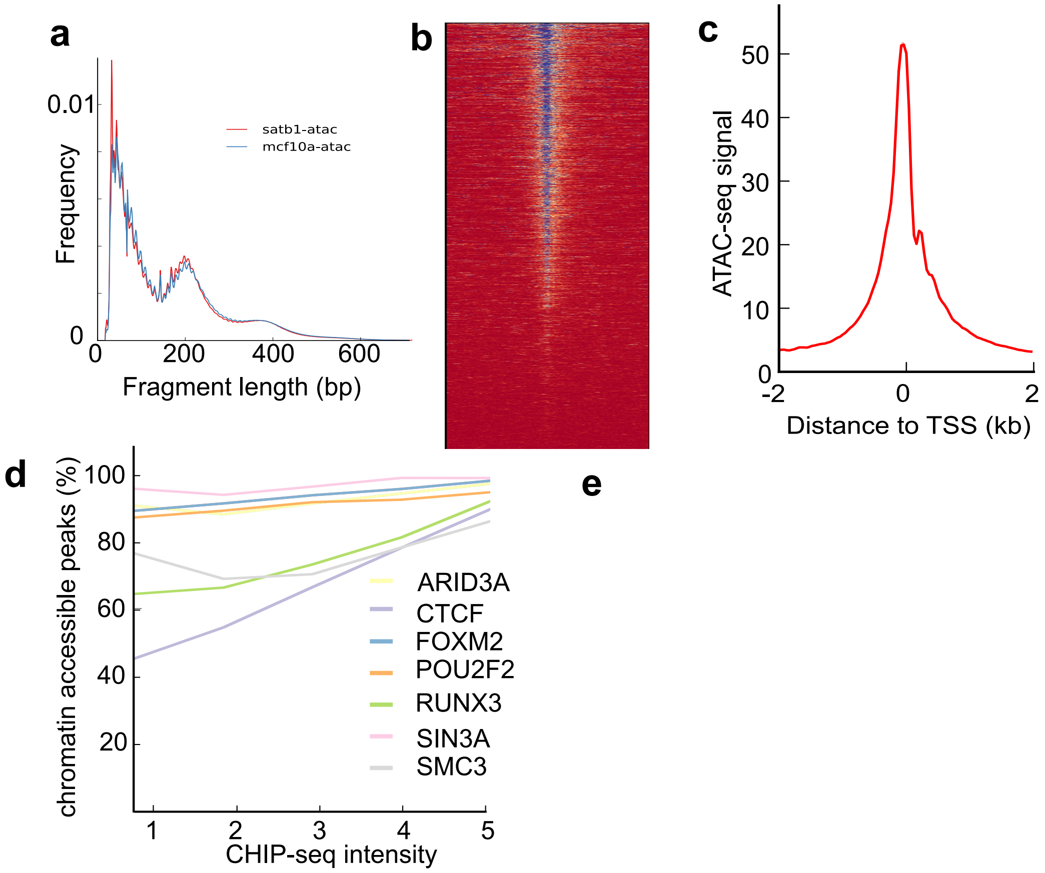

Supplementary Figure 6. Satb1 ATAC-seq features. a. Fragment length distribution of ATAC-seq reads in native MCF10A cells and cells expressing exogenous Satb1 show that most reads can be accounted for by nucleosome free regions and mono-nucleosomes. b. Heat map showing ATAC-seq reads stacked around a 4kb window on either side of Transcription start sites in MCF10A genome. c. The average of signals shown in b. d. Plot showing percentage of binding sites for 7 different transcription factors (see Methods for data source) that reside in transposase accessible regions of chromatin. Binding sites were classified into 5 quantiles based on increasing ChIP seq intensities (x axis).

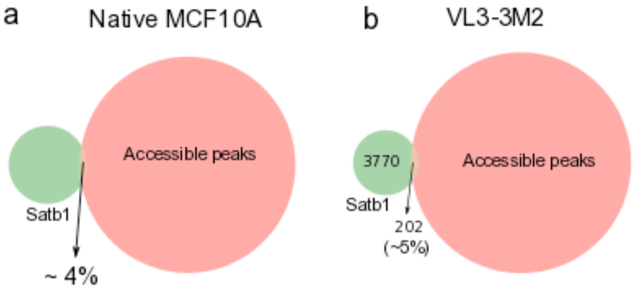

Supplementary Figure 7. Genome wide accessibility profile of Satb1 target sites in native MCF10A cells which lack Satb1 expression (a) and VL3-3M2 cells (b)

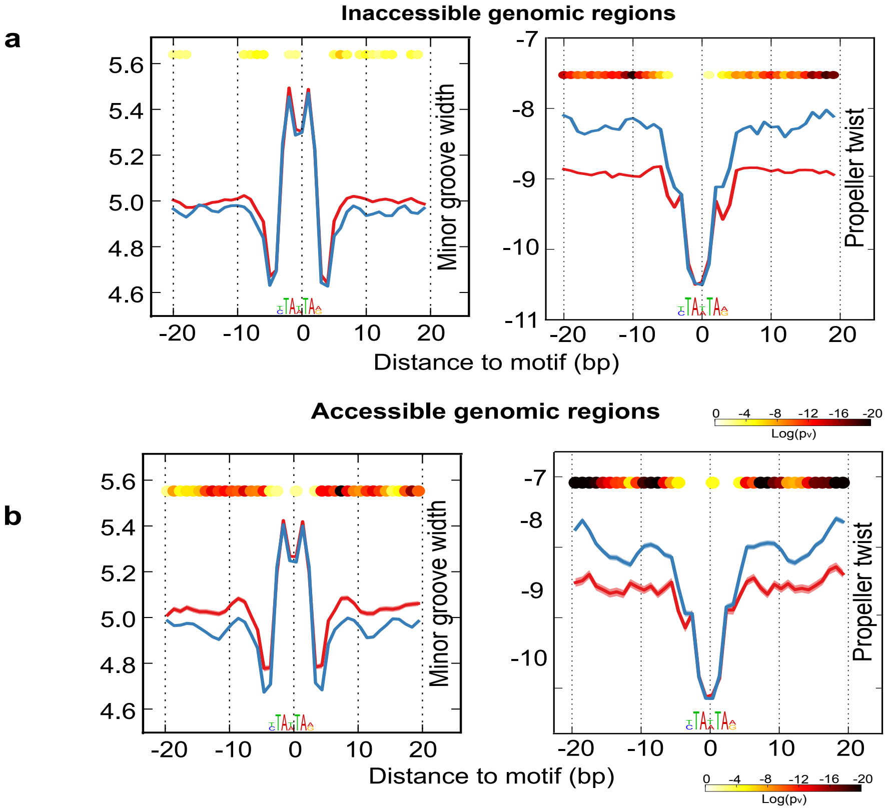

Supplementary Figure 8. Shape features of accessible and inaccessible Satb1 binding sites. a, b. Line plots showing the average of minor groove widths and propeller twists across tens of bases on either side of consensus sites bound by FL Satb1 in transposase inaccessible regions (a) and all transposase accessible regions (b). Blue line: Consensus motif without bound Satb1; Red line: Consensus motif with bound Satb1. Base-wise p-values calculated using Mann-Whitney U test, are shown with colored circles.

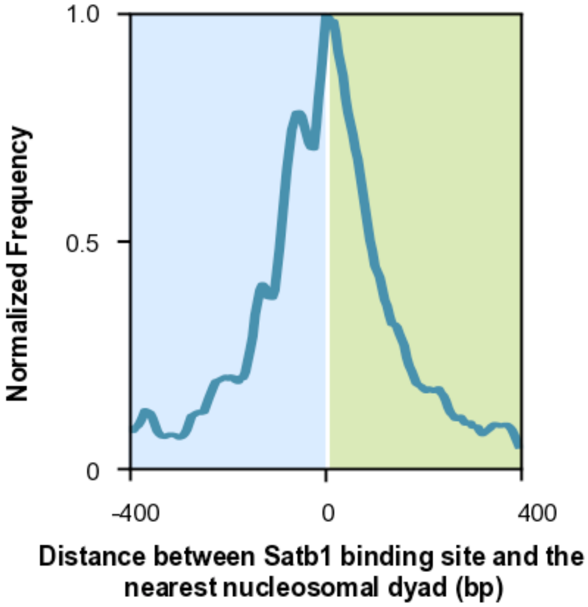

Supplementary Figure 9. Distribution of distances from nucleosomal dyad to Satb1 binding centers for all transposase accessible Sab1 binding sites in MCF-10A cells.

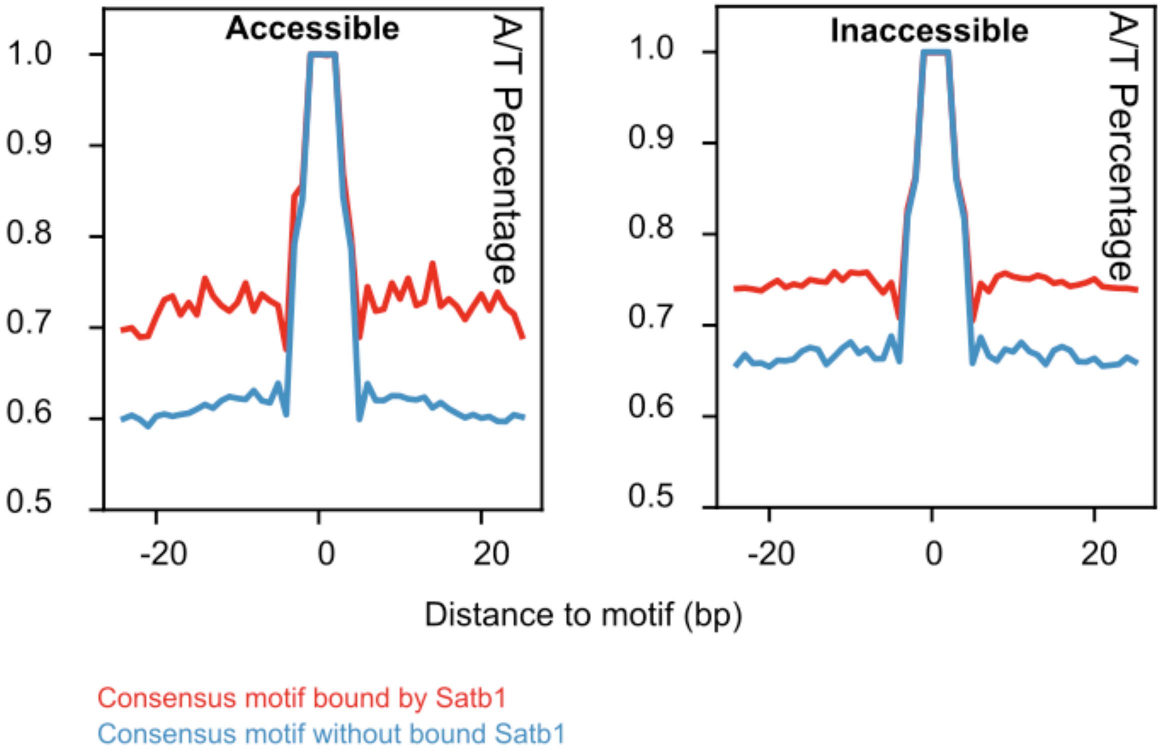

Supplementary Figure 10. Line plots showing average percent A/T content across 40 bp windows centered on consensus motifs bound by FL Satb1 in transposase inaccessible regions and all transposase accessible regions.

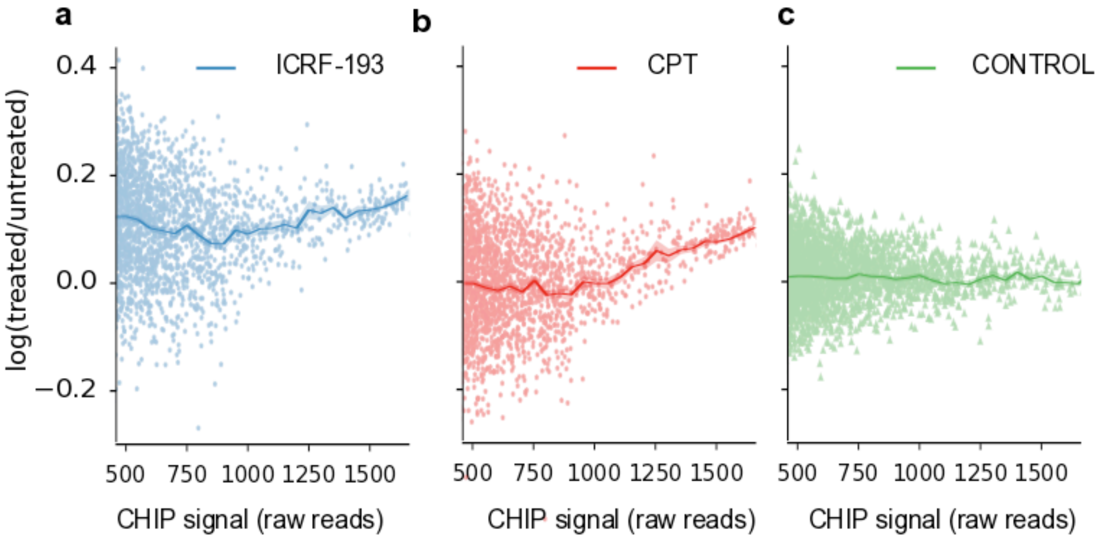

Supplementary Figure 11. Satb1 shows improved binding under enhanced negative torsional stress. Scatter plots corresponding to main text figure 4a**.**

Satb1 ChIP-seq signal ratio of “MCF-10A+Satb1” cells treated with Topo I or II inhibitors, ICRF-193 (blue) and CPT (red) respectively over untreated “MCF-10A+Satb1” cells, plotted against increasing raw ChIP-seq reads. The control line shows the ratio between two wild-type replicates. Error envelopes represent the standard error of mean.

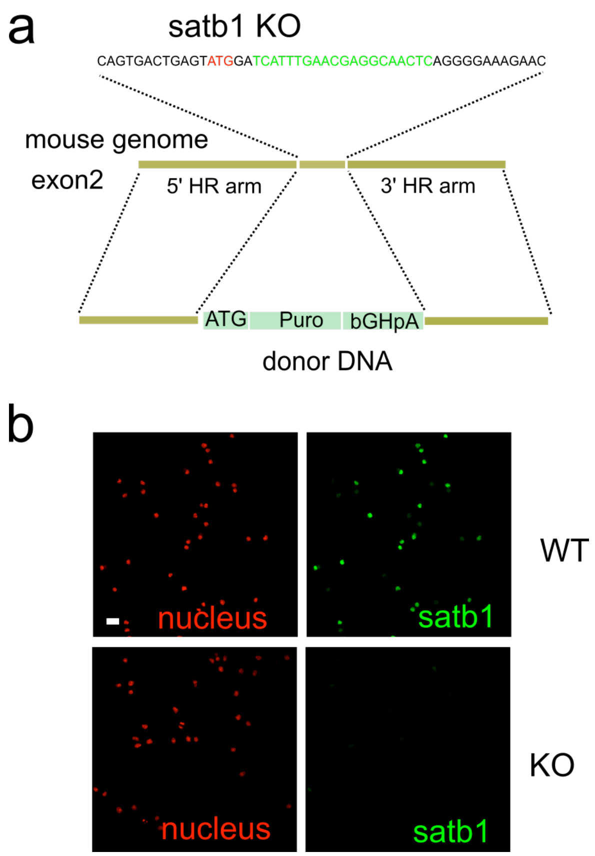

Supplementary Figure 12. CRISPR/ Cas9 based Satb1 knockout in VL3-3M2 cells. a. Schematic representation of CRISPR/Cas9 based knockout of native Satb1 in VL3 3M2 cells. b. Surveyor assay for verifying double strand breaks generated by sgRNA for Satb1 knockout. c. Verification of Satb1 knockout using immunofluorescence. Scale bar: 20μm

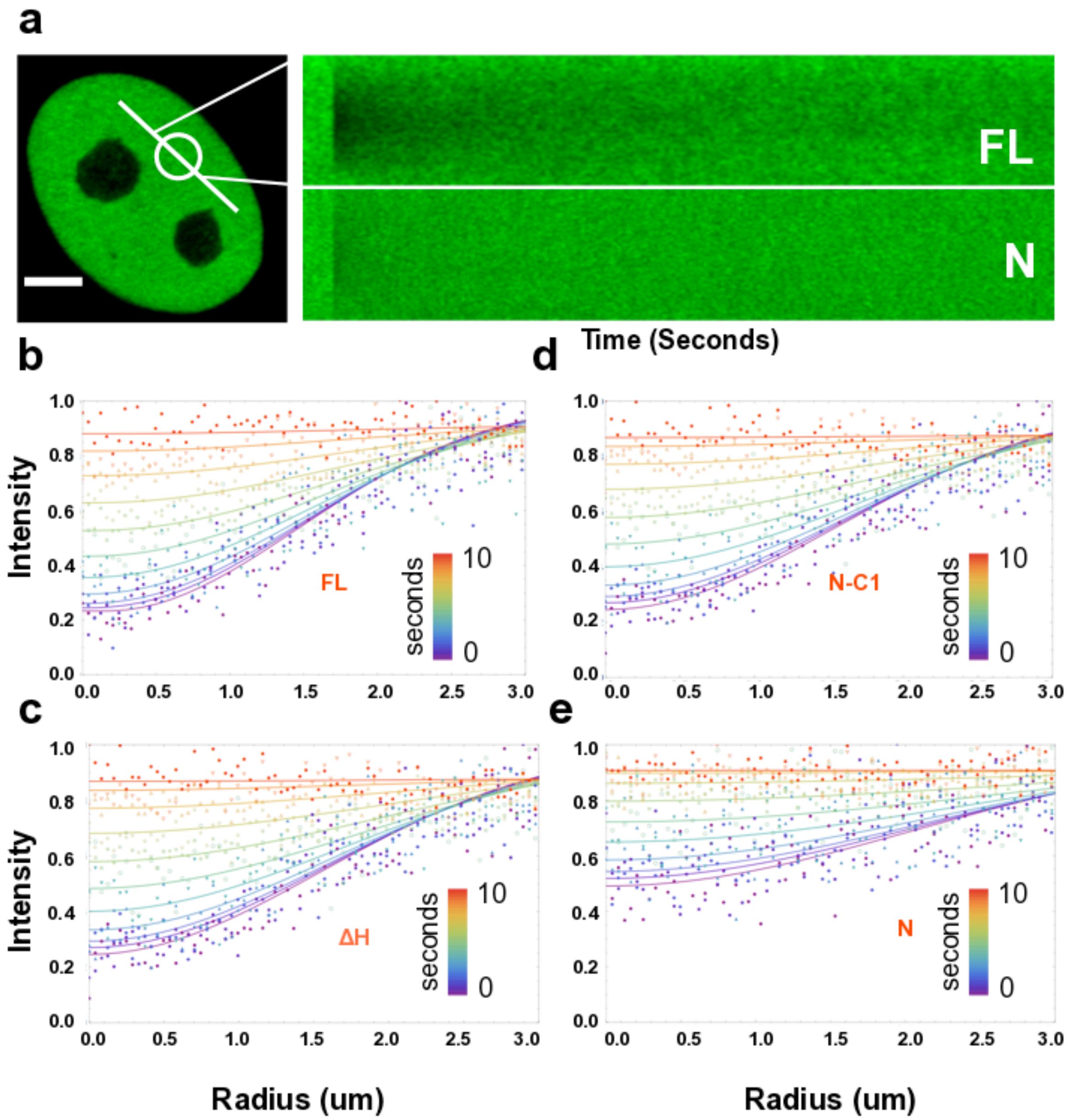

Supplementary Figure 13. Spatiotemporal FRAP analysis of Satb1 dynamics in MCF10A nucleus.
a. Examples of time vs fluorescence intensity carpets fluorescence bleaching and recovery along scan lines bisecting a bleach spot in MCF10A cells expressing exogenous eGFP fusions of FL Satb1 and the domain truncation mutant N (Scale bar is 5 μm).
b-e. The best fits of representative recovery profiles for FL (b), ∆HD (c), N-C1 (d) and N (e) are displayed at select times, with the color bar representing time in seconds.

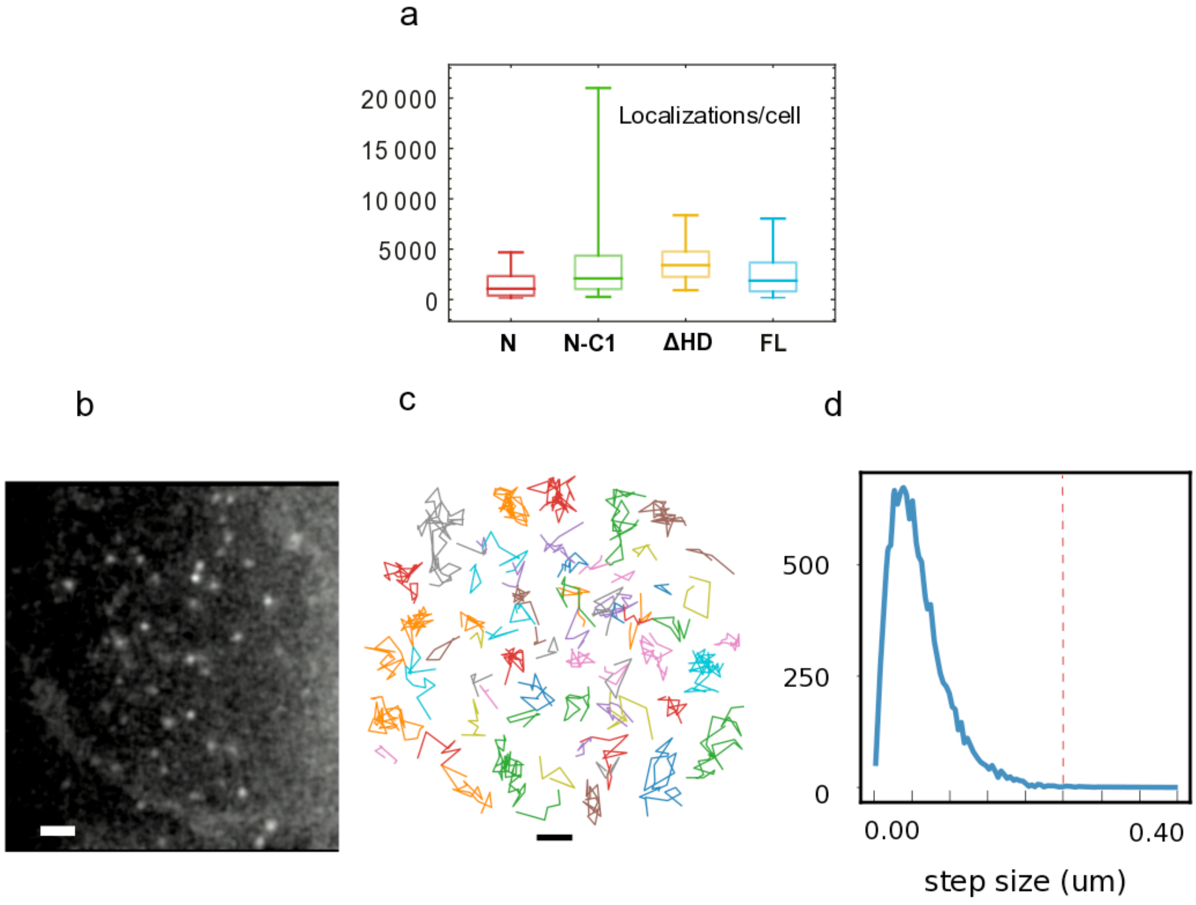

Supplementary Figure 14. Single molecule analysis of Satb1 and histone H2B-eGFP$_{4X}$ in the nucleus**.**
a. Satb1 single molecule localization distribution. All four Satb1 constructs (FL, D, N-C1 and ΔHD) have very similar numbers of total localizations, indicating comparable expression, imaging and tracking conditions. In box plot, the whisker shows the 1.5* IQR (interquartile range) over the 3rd quartile (top whisker) or below 1st quartile (lower whisker). Center line represents median. b. A Hilo TIRF image of an MCF-10A cell with leaky expression of H2B-eGFP$_{4X}$ showing diffraction limited spots of single H2B molecules (Scale bar is 2μm). c. Examples of H2B tracks (Scale bar is 200nm). d. Step size (frame to frame displacement) distribution of H2B molecules.

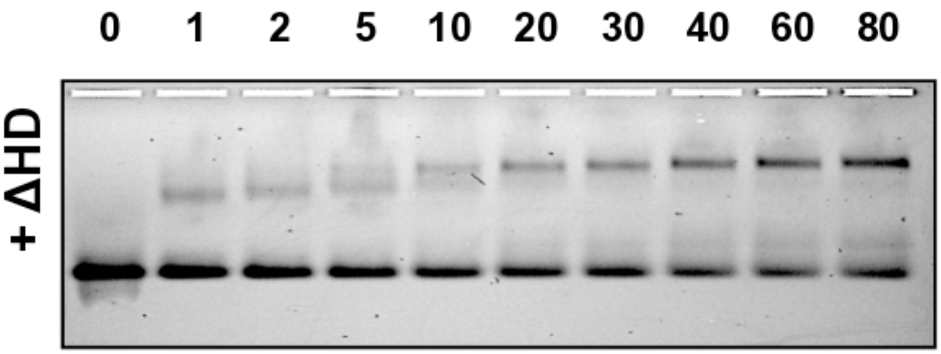

Supplementary Figure 15. 601A nucleosome-EMSA shows that Homeo domain truncation mutant ∆HD binds efficiently to nucleosome-core embedded consensus motif.

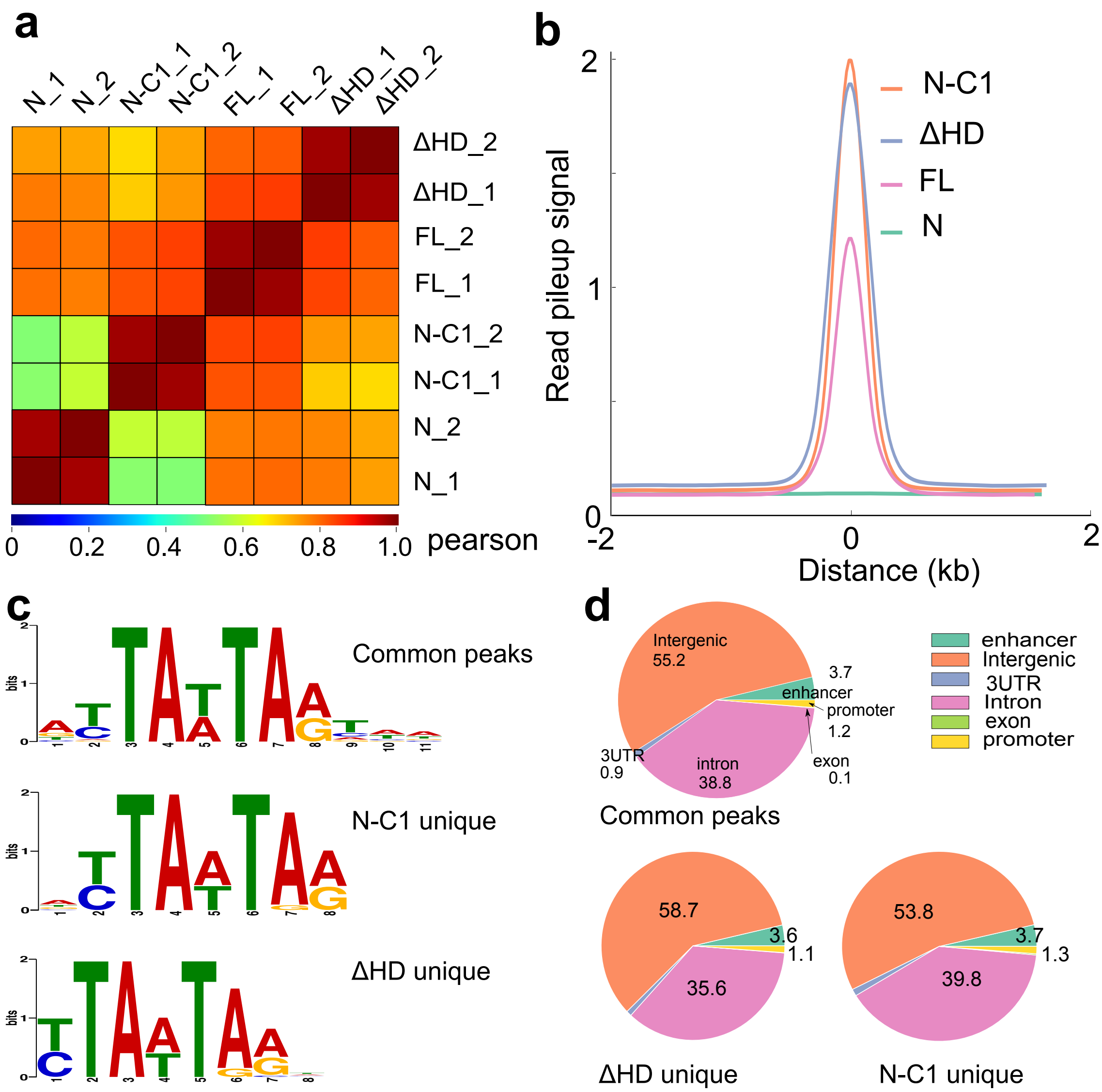

Supplementary Figure 16. Basic characterization of sites bound by different Satb1 domain mutations**.**
a. Heatmap of Pearson correlations between ChIP-seq replicates of Satb1 FL, ΔHD, N-C1, and N. b. Average plot of ChIP-seq signals shown in Figure 6a. c. Consensus motifs for binding sites that are common to FL, ΔHD and N-C1, and binding sites that are unique to N-C1 and ΔHD respectively. d. Genome feature annotation of binding sites that are common to FL, ΔHD and N-C1, and binding sites that are unique to N-C1 and ΔHD respectively.

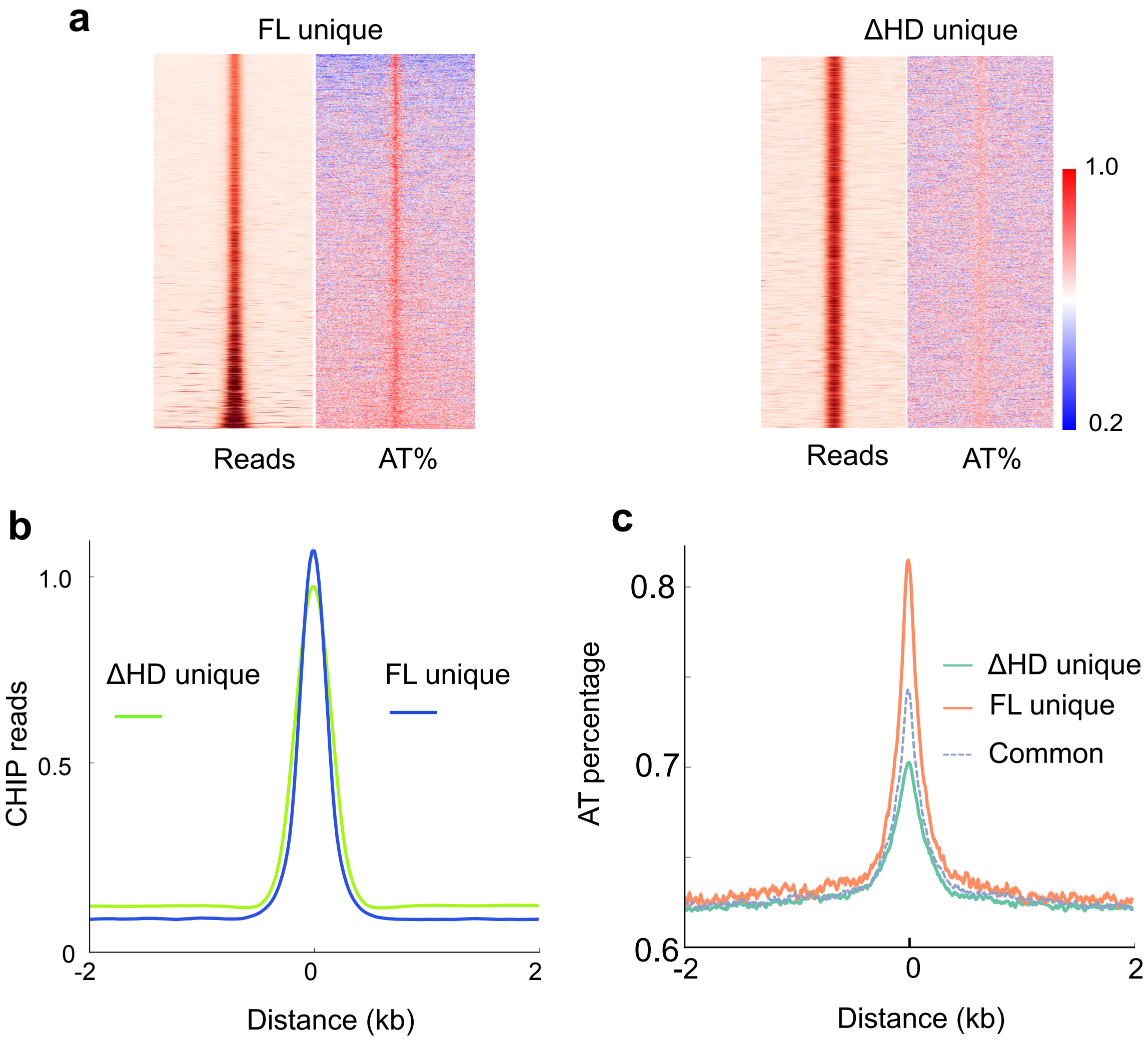

Supplementary Figure 17. Comparison of binding sites bound exclusively by FL Satb1 and ΔHD. a. Heatmap of ChIP-seq signals (read pileups) (left column) and AT percentage (right column) of binding sites unique to FL and ΔHD. b, c. Average plot of ChIP-seq signals (b) and AT percentage (c) along a 4kb window across the binding sites that are exclusively bound by FL Satb1 or ΔHD.

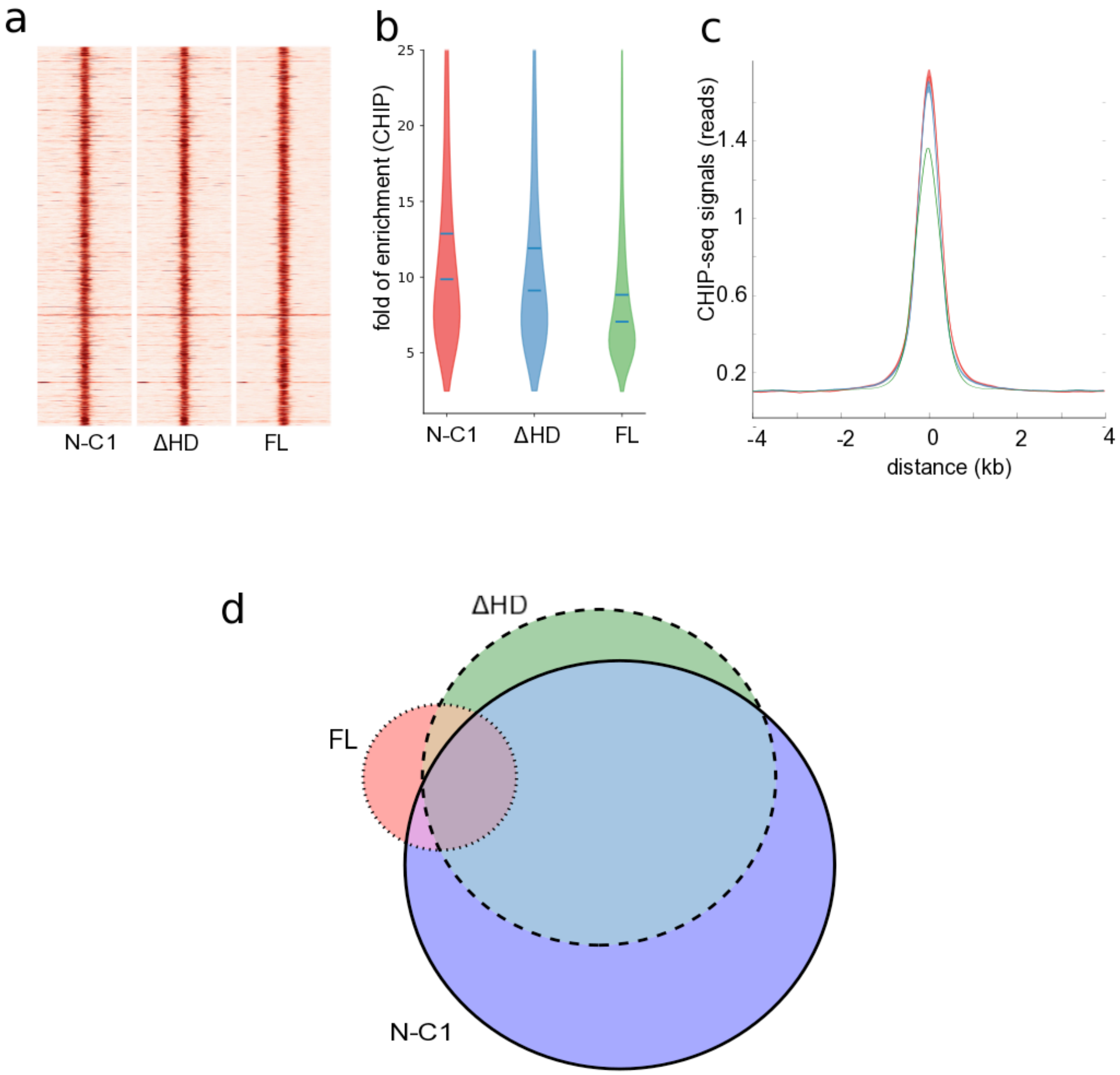

Supplementary Figure 18. Homeo domain is dispensable for high affinity binding in VL3-3M2
a. Heatmap of ChIP-seq signals (read pileups) along a 4kb window centered on binding peaks of different Satb1 domain constructs. b. Violin plots showing the distribution of the peak signal strengths for 3 different Satb1 constructs (peak strengths are shown as horizontal lines: mean (upper line) and median (lower line)). c. Average plot of ChIP-seq signals along a 4kb window across the binding sites. d. Venn diagram showing binding site overlap between all 3 domain constructs.

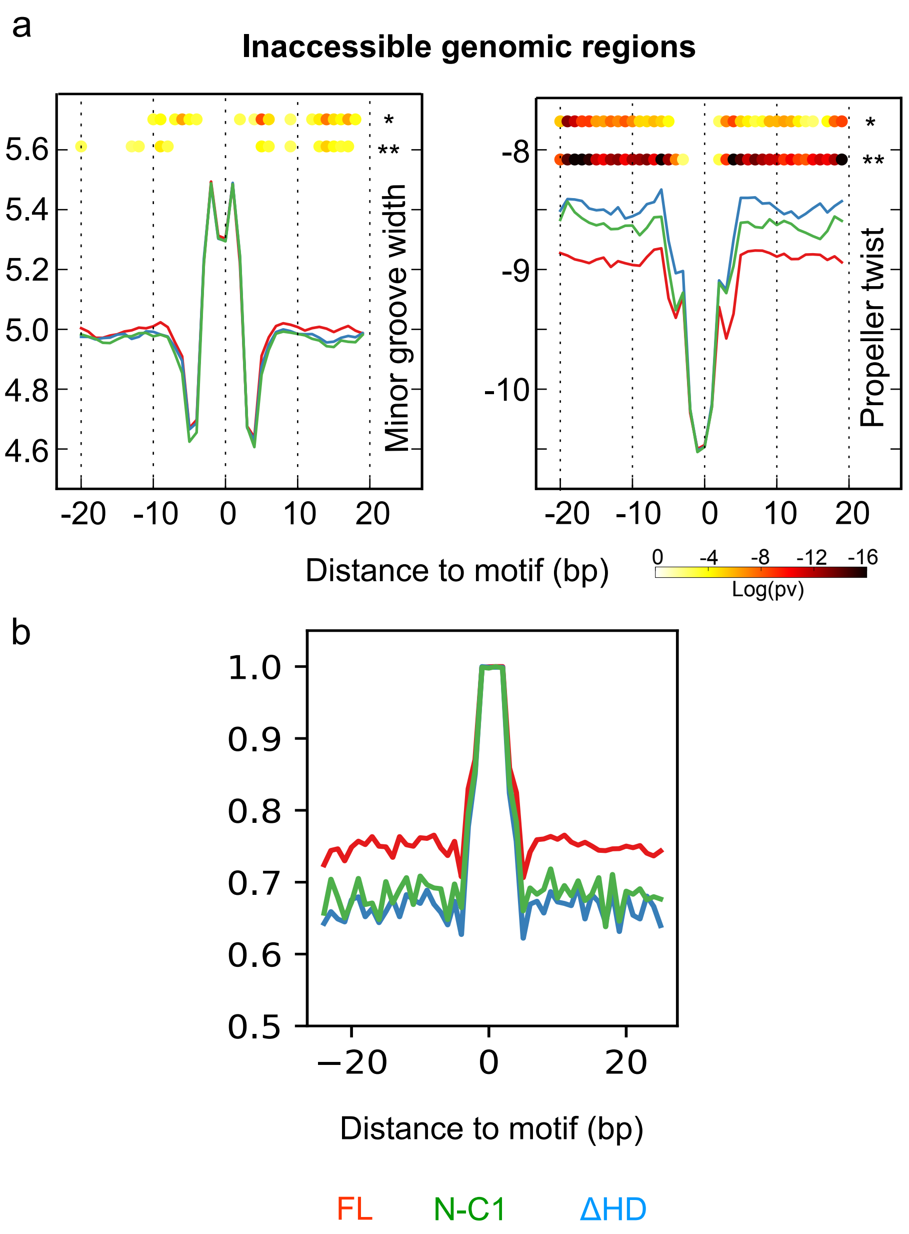

Supplementary Figure 19. Shape features of sites exclusively bound by Satb1 domain mutants in inaccessible chromatin.

Line plots showing the average of minor groove width, propeller twists (a) and A/T percentages (b) across tens of bases on either side of sites exclusively bound by FL Satb1 and sites bound exclusively by N-C1 and ΔHD in transposase inaccessible regions (* basewise p-values for FL vs. N-C1; ** basewise p-values for FL vs ΔHD, calculated based on Mann-Whitney U test)
