# Supplementary figures and images for: Satb1 integrates DNA binding site geometry and torsional stress to differentially target nucleosome-dense regions
Source: Nat Commun. 2019 Jul 19;10:3221. doi: 10.1038/s41467-019-11118-8 (PMC6642133; doi:10.1038/s41467-019-11118-8)

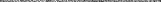

Supplement: Supplementary file 8 — Supplementary Code 1 [file 41467_2019_11118_MOESM8_ESM.zip › Custom codes/mathematica_script_spatio_temproal_frap /sample.tif]

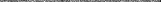

Supplement: Supplementary file 8 — Supplementary Code 1 [file 41467_2019_11118_MOESM8_ESM.zip › Custom codes/mathematica_script_spatio_temproal_frap /sample_control.tif]

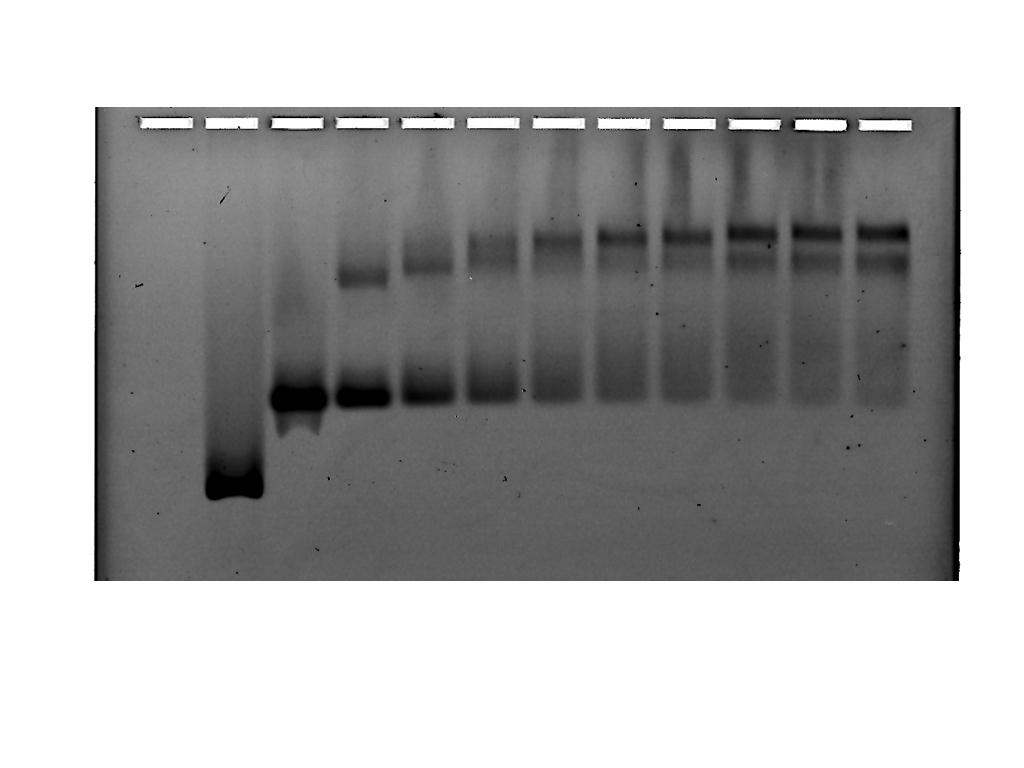

Supplement: Supplementary file 10 — Source Data [file 41467_2019_11118_MOESM10_ESM.zip › Source data/EMSA gels/main text full gel FL.001.jpeg]

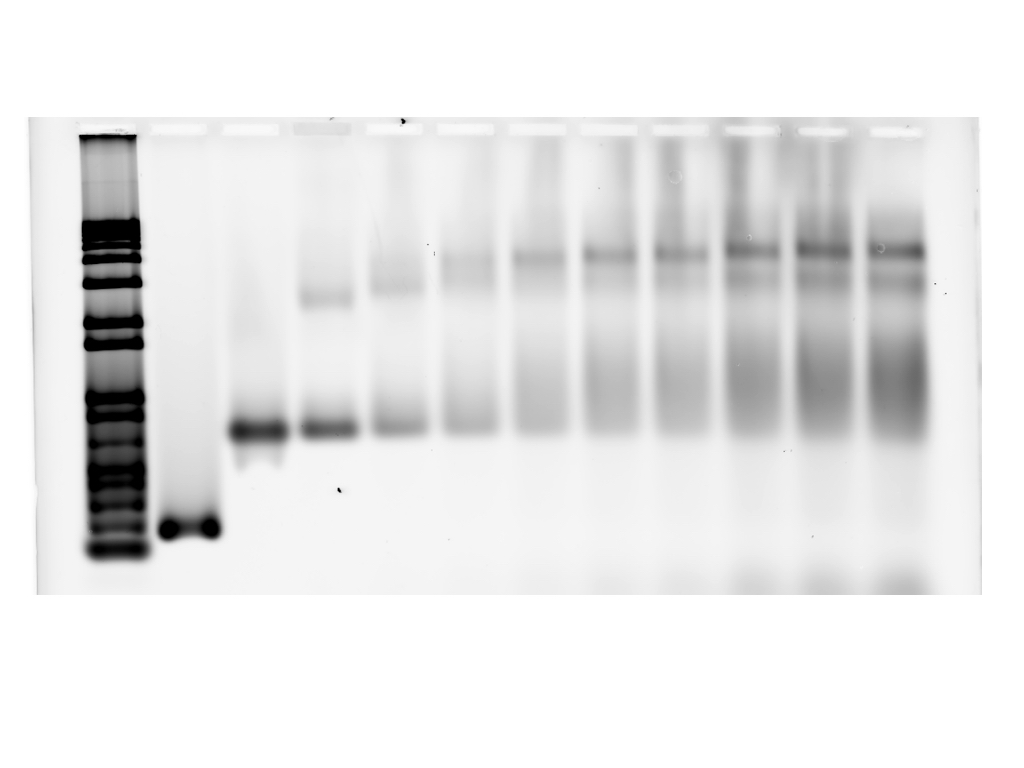

Supplement: Supplementary file 10 — Source Data [file 41467_2019_11118_MOESM10_ESM.zip › Source data/EMSA gels/main text full gel_FL_sybr green.jpeg]

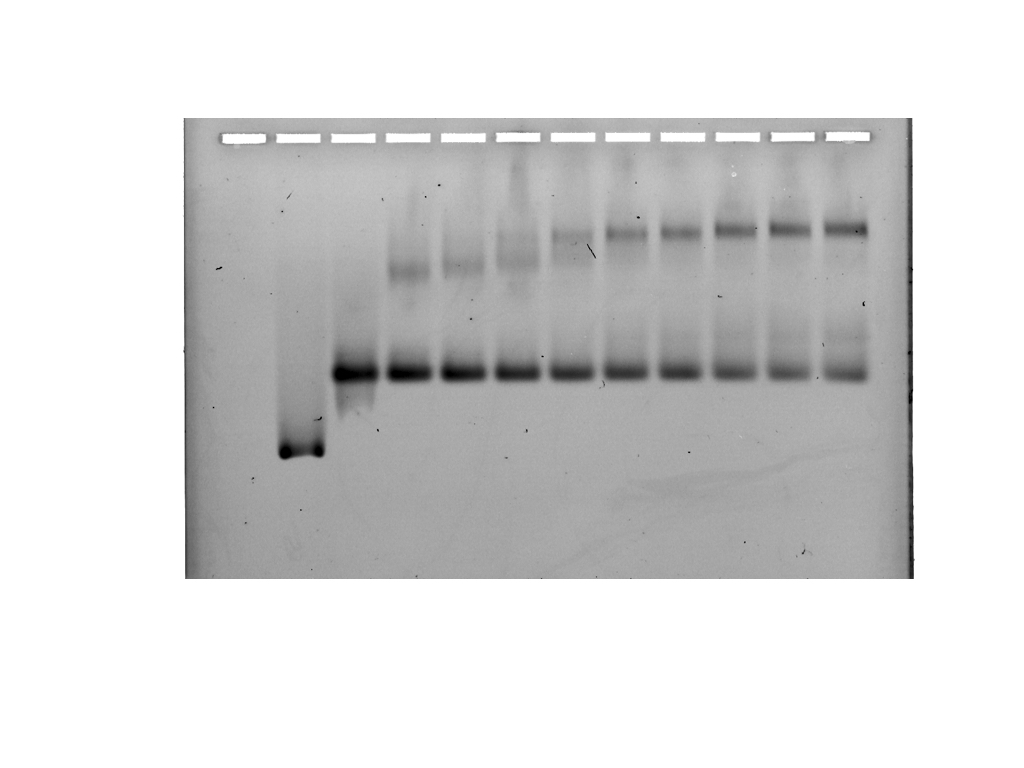

Supplement: Supplementary file 10 — Source Data [file 41467_2019_11118_MOESM10_ESM.zip › Source data/EMSA gels/Supplementary Figure Del HD full gel.001.jpeg]
