# Supplementary material for: Satb1 integrates DNA binding site geometry and torsional stress to differentially target nucleosome-dense regions
Source: Nat Commun. 2019 Jul 19;10:3221. doi: 10.1038/s41467-019-11118-8 (PMC6642133; doi:10.1038/s41467-019-11118-8)
Supplement: Supplementary file 9 — Reporting Summary [file 41467_2019_11118_MOESM9_ESM.pdf]

nature research

Corresponding author(s): Jan Liphardt

Reporting Summary

Nature Research wishes to improve the reproducibility of the work that we publish. This form provides structure for consistency and transparency in reporting. For further information on Nature Research policies, see Authors & Referees and the Editorial Policy Checklist.

Statistical parameters

When statistical analyses are reported, confirm that the following items are present in the relevant location (e.g. figure legend, table legend, main text, or Methods section).

n/a	Confirmed	
☐	☒	The exact sample size (n) for each experimental group/condition, given as a discrete number and unit of measurement
☐	☒	An indication of whether measurements were taken from distinct samples or whether the same sample was measured repeatedly
☐	☒	The statistical test(s) used AND whether they are one- or two-sided<br>*Only common tests should be described solely by name; describe more complex techniques in the Methods section.*
☒	☐	A description of all covariates tested
☒	☐	A description of any assumptions or corrections, such as tests of normality and adjustment for multiple comparisons
☐	☒	A full description of the statistics including central tendency (e.g. means) or other basic estimates (e.g. regression coefficient) AND variation (e.g. standard deviation) or associated estimates of uncertainty (e.g. confidence intervals)
☐	☒	For null hypothesis testing, the test statistic (e.g. F, t, r) with confidence intervals, effect sizes, degrees of freedom and P value noted<br>*Give P values as exact values whenever suitable.*
☒	☐	For Bayesian analysis, information on the choice of priors and Markov chain Monte Carlo settings
☒	☐	For hierarchical and complex designs, identification of the appropriate level for tests and full reporting of outcomes
☒	☐	Estimates of effect sizes (e.g. Cohen's d, Pearson's r), indicating how they were calculated
☐	☒	Clearly defined error bars<br>*State explicitly what error bars represent (e.g. SD, SE, CI)*

Our web collection on statistics for biologists may be useful.

Software and code

Policy information about availability of computer code

Data collection	Images were acquired on Zeiss LSM 700 using the Zen imaging software, and single molecule HILo TIRF images were acquired with a custom-written application. All NGS sequences were obtained using Illumina sequencer at the Functional Genomics Facility at Stanford as described in the manuscript.
Data analysis	Image processing were performed by Imaris and custom-code in python and mathematica. Analysis of particle trajectories was done using a variety of custom-written codes and open-source software widely used in the community (see Methods section for specific details). Detectable particles in each frame were first identified by performing a Laplacian of Gaussian filter and then thresholding the filtered image based on intensity. Spatiotemporal FRAP analysis was done using custom written codes in mathematica. In NGS data analysis, various open source packages were used, including Bowtie for genome alignment, bedtools,samtools, and MACS2 for peak calling. Analysis and plotting were done using custom python codes using matplotlib.

For manuscripts utilizing custom algorithms or software that are central to the research but not yet described in published literature, software must be made available to editors/reviewers upon request. We strongly encourage code deposition in a community repository (e.g. GitHub). See the Nature Research guidelines for submitting code & software for further information.

Data

Policy information about availability of data

All manuscripts must include a data availability statement. This statement should provide the following information, where applicable:

- Accession codes, unique identifiers, or web links for publicly available datasets
- A list of figures that have associated raw data
- A description of any restrictions on data availability

NGS data will be uploaded to public database SRA/GEO, and available to public. Other data and custom codes will be available upon request.

Field-specific reporting

Please select the best fit for your research. If you are not sure, read the appropriate sections before making your selection.

☒ Life sciences ☐ Behavioural & social sciences ☐ Ecological, evolutionary & environmental sciences

For a reference copy of the document with all sections, see nature.com/authors/policies/ReportingSummary-flat.pdf

Life sciences study design

All studies must disclose on these points even when the disclosure is negative.

Sample size	In general we severely oversample for each experiment. For single particle tracking experiments, we acquired data from more than 20 cells per condition with total tracks between 2000-8000. Spatiotemporal FRAP experiments were performed twice in the same cell and for each condition data were acquired from 20 to 30 cells. Where relevant, all curves/numbers/plots are given with standard deviations or standard errors of the mean (see main text and figures). For all genomics experiments (CHIPseq, ATAC-seq, TMP-seq) experiments were performed in duplicates and consistency of results was verified for each experiment type.
Data exclusions	NA
Replication	For each imaging based experiment data were collected from mutiple/10s of cells. For sequencing data, replicates for each experiment were conducted and verified that the results and finding is reproducible.
Randomization	NA
Blinding	NA

Reporting for specific materials, systems and methods

Materials & experimental systems

n/a	Involved in the study	
☒	☐	Unique biological materials
☐	☒	Antibodies
☐	☒	Eukaryotic cell lines
☒	☐	Palaeontology
☒	☐	Animals and other organisms
☒	☐	Human research participants

Methods

n/a	Involved in the study	
☐	☒	ChIP-seq
☒	☐	Flow cytometry
☒	☐	MRI-based neuroimaging

Antibodies

Antibodies used	GFP antibody from Abcam ab290 for genomics, Satb1 antibody from abcam for immunofluorescence as well as for genomics
Validation	GFP antibody used for CHIP-seq are CHIP grade from Abcam. Antibodies are verified by comparing our immunofluorescence data with published immunofluorescence data.

Eukaryotic cell lines

Policy information about cell lines

Cell line source(s)	MCF10A and Vl3 cells obtained from other lab
Authentication	None of these cell lines have been authenticated in our lab. However it should be noted that cells were obtained from reliable well established labs and cell phenotypes were identical to published results.
Mycoplasma contamination	No mycoplasma testing was performed.
Commonly misidentified lines (See ICLAC register)	No commonly misidentified cell lines were used.

ChIP-seq

Data deposition

☒ Confirm that both raw and final processed data have been deposited in a public database such as GEO.

☒ Confirm that you have deposited or provided access to graph files (e.g. BED files) for the called peaks.

Data access links *May remain private before publication.*	To review GEO accession GSE123292:<br>Go to<br>https://www.ncbi.nlm.nih.gov/geo/query/acc.cgi?acc=GSE123292<br>Enter token gncpamycdxyhxuz into the box
Files in database submission	Fastq files for MCF10A_FL, dHD, NC1, N terminal and inputs. MACS2 peak calling narrowPeak files for FL, dHD, and NC1, and bigwig tracks for MCF10A_N
Genome browser session (e.g. UCSC)	NA

Methodology

Replicates	replicates were performed, with very consistent results (correlation coefficient r>0.997 as described in the manuscript)
Sequencing depth	CHIP libraries were sequenced to between 20-50 millian reads 75bp in length
Antibodies	CHIP grade GFP antibody ab290 from abcam. The Satb1 antibody is from abcam
Peak calling parameters	sequence aligned to human genome hg19 and mouse genome mm10 with default parameters of Bowtie; Peak calling uses default parameters in MACS2, FDR=0.01. CHIP input DNA was used as peak calling controls.
Data quality	reads have good quality, e.g majority read score > 30, alignment rate>90%, and >20000 peaks with FDR<5% and 5 fold enrichment.
Software	FASTQC was used for quality control and assessment. Data were trimmed with AdaptCut 1.18, and aligned with Bowtie2. Peak calling uses MACS2.
