# Supplementary material for: Satb1 integrates DNA binding site geometry and torsional stress to differentially target nucleosome-dense regions
Source: Nat Commun. 2019 Jul 19;10:3221. doi: 10.1038/s41467-019-11118-8 (PMC6642133; doi:10.1038/s41467-019-11118-8)
Supplement: Supplementary file 8 — Supplementary Code 1 [file 41467_2019_11118_MOESM8_ESM.zip › Notes for raw data files and custom codes - Copy.doc]

Notes for raw data files and custom codes

The analysis and plotting were mostly done using various packages in python, and matplotlib for plotting

**Figure 2**

a-c: CHIP-seq reads and peakcalling files are deposited to GEO. use deeptools for heatmap plot

d. annotation use Homer software based on CHIP-seq peak file, deposited to GEO

e-g. motif search use FIMO and MEME tools; analyzed data for plotting provided in the Excel file. Data file named FL_peaks_RB_mo1_cm.bed included, using custom code=21 in custom_code.py file. Figure 2g uses custom_code.py code=22

h-i. ucsc browser for displaying bigwig files, which are generated from chip-seq BAM files, using ucsc tool: bedGraphtoBigWig

**Figure 3**

a,c,d: heatmap and line plots are generated using deeptools, and calculation based on the matrix; atac-seq data are deposited at GEO

b. venn diagrams are generated by python package matplotlib_venn, chip-seq peak files and atac-seq peak file are deposited at GEO

e-g: nucleosome position maps for both ctcf in mcf10a, and satb1 in mcf10a and mcf10a expressing satb1 are provided (files: satb1_mcf10a_satb1+nucpos.bed, satb1_mcf10a_nucpos.bed, ctcf_mcf10a_nucpos.bed), also including custom codes, code=31

i,j; all raw data for shape analysis are included in the DNAshape folder (subfolders: satb1_open, satb1_nfr, satb1_close, satb1_motif_unbound_nfr, satb1_motif_unbound_open).

**Figure 4**

a, raw data Satb1 chip-seq peak files for treated and untreated, and TMP-seq signal files (bigwig) are deposited to GEO

b. the line plot data are included in the Excel fle

c. generated from ucsc genome browser

d-e, generated from 6 TMP_seq track files, TMP-seq in MCF10A, +CP treatment, +ICRF treatment, and MCF10A expressing Satb1 untreated and treated with CP and ICRF, files are deposited at GEO

**Figure 5**

a, b. All analysis were done using in house TMP seq (have been uploaded to GEO) and publicly available CHIP-seq data (For GEO accession numbers see Methods)

**Figure 6**

Spatiotemporal FRAP in VL3-3M2 raw data have been included in the raw data folder.

Custom code written in Mathematica for Spatiotemporal FRAP analysis is also included.

**Figure 7**

single molecules tracking data are included in folder: SM_track_duration (4 satb1 mutants: FL, dHD, NC1, and N)

g-i. generated from sinngle molcule track duration files (included in single molecule tracks folder). The files are compressed and included for all 4 satb1 mutants.

The fitted parameters are listed in the Excel file

**Figure 8**

a, heatmap are generated using python package deeptools. raw data are deposited to GEO

b,c,d: generated using CHIP-seq narrowPeak files, deposited to GEO; violin plots are done with seaborn package in python, venn diagrams are obtained with matplotlib_venn package

e-g, generated similarly as described above. Raw data are deposited to GEO

h, data are included in Excel file.

i: DNA shape data are included (in DNAshape folder, subfolders NC1_open and dHD_open).

**Supplementary Figure 3**

correlation using deeptools plotCorrelation, based on CHIP-seq data deposited to GEO

**Supplementary Figure 4**

vl3 chip-seq data are deposited to GEO

**Supplementary Figure 5**

Using publicly available dataset

**Supplementary Figure 6**

Atac-seq data are deposited to GEO

**Supplementary Figure 7**

Atac-seq data are deposited to GEO

**Supplementary Figure 8, 10, 19:** DNA shape data are included as described above under Figure 3

**Supplementary Figure 13:** Spatiotemporal FRAP in MCF10A raw data have been included in the raw data folder.

Custom code written in Mathematica for Spatiotemporal FRAP analysis is also included.

**Other genomic raw data (CHIP-seq data, ATAC-seq data) including all TMP-seq data are included in the raw data folder or have been deposited to GEO.**

The full EMSA gels corresponding to Main Text Figure 3h and Supplementary Figure 15 have been included.
